# Quantifying Stochastic Noise in Cultured Circadian Reporter Cells

**DOI:** 10.1371/journal.pcbi.1004451

**Published:** 2015-11-20

**Authors:** Peter C. St. John, Francis J. Doyle

**Affiliations:** 1 Department of Chemical Engineering, University of California, Santa Barbara, Santa Barbara, California, United States of America; 2 Biosciences Center, National Renewable Energy Laboratory, Golden, Colorado, United States of America; 3 Paulson School of Engineering and Applied Sciences, Harvard University, Cambridge, Massachusetts, United States of America; University of Illinois at Urbana-Champaign, UNITED STATES

## Abstract

Stochastic noise at the cellular level has been shown to play a fundamental role in circadian oscillations, influencing how groups of cells entrain to external cues and likely serving as the mechanism by which cell-autonomous rhythms are generated. Despite this importance, few studies have investigated how clock perturbations affect stochastic noise—even as increasing numbers of high-throughput screens categorize how gene knockdowns or small molecules can change clock period and amplitude. This absence is likely due to the difficulty associated with measuring cell-autonomous stochastic noise directly, which currently requires the careful collection and processing of single-cell data. In this study, we show that the damping rate of population-level bioluminescence recordings can serve as an accurate measure of overall stochastic noise, and one that can be applied to future and existing high-throughput circadian screens. Using cell-autonomous fibroblast data, we first show directly that higher noise at the single-cell results in faster damping at the population level. Next, we show that the damping rate of cultured cells can be changed in a dose-dependent fashion by small molecule modulators, and confirm that such a change can be explained by single-cell noise using a mathematical model. We further demonstrate the insights that can be gained by applying our method to a genome-wide siRNA screen, revealing that stochastic noise is altered independently from period, amplitude, and phase. Finally, we hypothesize that the unperturbed clock is highly optimized for robust rhythms, as very few gene perturbations are capable of simultaneously increasing amplitude and lowering stochastic noise. Ultimately, this study demonstrates the importance of considering the effect of circadian perturbations on stochastic noise, particularly with regard to the development of small-molecule circadian therapeutics.

## Author Summary

As most organisms exist in an environment that changes predictably with a 24-hour period, highly optimized genetic circuits turn on and off the production of key regulatory proteins to anticipate the day/night cycle. In humans, the demands of a modern society have required that we deviate from this evolutionarily prescribed sleep and feeding schedule, resulting in increased long-term risks of metabolic disease. There is therefore a desire to find pharmacological treatments that would restore the normal functioning of our circadian clock despite irregular behavioral schedules. One aspect of these treatments that is often overlooked in searching for candidate drugs is how these treatments might affect the accuracy of the circadian timing system. Recording the time of each cell is possible but difficult; as a result single-cell approaches cannot be scaled up to high-throughput searches. In this paper, we show that it is possible to estimate how much the noise of a system has changed by looking only at the averaged protein production of an entire population of cells. Such an approach allows us to analyze prior data from high-throughput screens, and show that the natural clock has been highly optimized to be both accurate and high amplitude.

## Introduction

Circadian rhythms are daily changes in gene expression and physiology that persist even in the absence of external environmental cues [1]. In mammals, such rhythms are organized in a hierarchical fashion: at the tissue-level, the brain’s suprachiasmatic nucleus (SCN) serves as the master pacemaker and keeps circadian oscillations in peripheral tissues in phase with the light-dark cycle. In the SCN, cell-to-cell coupling keeps individual cells in tight synchrony [2], while coupling between circadian oscillations in peripheral tissues *in vivo* or cultured reporter cells *in vitro* is thought to be very weak or absent entirely [3, 4]. Within each tissue, cellular-level rhythms in gene transcription are generated by a large network of interacting gene regulatory elements, in which time-delayed transcription-translation negative feedback gives rise to sustained oscillations [5]. The robust oscillation of circadian factors has been linked to metabolic health [6], since rhythms compromised by gene knockout [7] or irregular feeding schedules [8] result in an increased risk of metabolic disease. Additionally, as the amplitude of circadian transcription can be affected by lifestyle variables such as diet, age, or work schedule, there has been recent interest in developing pharmacological strategies for increasing the amplitude of circadian cycles in metabolic tissues [9].

A detailed understanding of the underlying transcriptional mechanisms is essential for the development of circadian therapeutics to be successful. The functional roles of different genes in circadian regulation have traditionally been studied using behavioral-level data and genetic knockout experiments [10]. Bioluminescence-based cellular circadian reporters offer a more direct view of the gene regulatory network [11] and are amenable to high-throughput screens, allowing genome-wide exploration into factors that affect circadian rhythmicity [12]. Additionally, cultured circadian reporter cells allow the change in transcriptional amplitude following a perturbation to be quantified. This additional parameter has proven useful in differentiating between perturbations with the same effect on period [13] and has aided the search for small-molecule therapeutics to boost clock amplitude [9].

Bioluminescence rhythms at the cell culture or tissue-level are the result of the collective behavior of thousands of individual cells. Transcription at the single-cell level is strongly affected by *intrinsic* cellular noise, caused by the low molecular counts of the mRNA and protein species involved. As a result, bioluminescence traces of individual cells are stochastic, with significant variability in both amplitude and period length from cycle to cycle [14]. In addition to intrinsic noise, circadian oscillations are also affected by *extrinsic* noise sources. Extrinsic noise results from heterogeneity between cells, such as differences in size or physical environment, leading to differences on a cell-to-cell basis in expected period and amplitude. The effects of noise in biological systems has been well-studied, and relative amounts of intrinsic and extrinsic noise can be identified through carefully designed single-cell experiments [15]. For circadian systems, intrinsic noise has been suspected to play a larger role: a single cell’s variability in period from cycle-to-cycle is larger than the variability in mean period length between cells [2]. However, both sources of noise have an effect on population-level rhythms: in cell cultures that lack cell-to-cell coupling, it has been shown that stochastic noise is manifested in damped oscillations at the population-level as individual oscillators gradually drift out of phase [14, 16]. This type of behavior has also been seen in other experimental systems, such as NF-κB signaling or yeast glycolytic oscillations [17, 18]. The amount of noise in system is therefore linked to the ability of tissue-level clocks to maintain high-amplitude rhythms.

Despite the averaging that occurs at the population-level, cell-autonomous stochastic noise plays an important role in determining the function of the circadian oscillator. Noise in circadian rhythms has long been considered an important factor in how circadian rhythms have evolved [19]. A recent study showed that stochasticity is critical to the population-level response to a neuropeptide and forms the basis for how the SCN entrains to light-mediated cues [20]. Additional studies have suggested that the basis of single-cell rhythmicity may depend on stochastic noise, as models of deterministically damped oscillators, when simulated stochastically, capture the noise characteristics seen in single-cell fibroblast data equally well as limit-cycle oscillators [21]. Despite the importance of single-cell stochasticity in circadian rhythms, measuring stochastic noise currently requires careful preparation, recording, and image processing of individual cells [22]. As a result, while circadian perturbations have been postulated to affect single-cell stochasticity [23], no study has experimentally quantified changes to stochastic noise as a result of a small molecule or genetic perturbation.

In this study, we demonstrate that changes to stochastic noise can be reliably inferred from the changes to the damping rate of population-level bioluminescence recordings of cultured circadian reporters. Our method assumes that oscillations in individual cells are both

- independent (no significant cell-to-cell coupling) [3, 4],
- and sustained (do not damp on a single-cell basis) [14, 16],

which have been shown to hold experimentally for cultured fibroblast cells. We demonstrate the validity and usefulness of such an approach on several types of circadian data. First, we show using single-cell fibroblast data that intrinsic stochastic noise is directly related to population-level damping. Next, we show that a small-molecule modulator is able to change damping rate in a dose-dependent fashion, and verify using a mathematical model that changes to intrinsic stochastic noise is a likely mechanism. Finally, we calculate the genome-wide effects of siRNA knockdown on overall stochastic noise, and demonstrate that population-level damping rate is independent of other circadian parameters, such as period or amplitude. Using this additional information, we show that circadian rhythms have likely evolved to an optimal point of high amplitude and low stochastic noise. Our results should prove especially important in the future search for small molecule circadian therapeutics, as it allows the effect of candidate drugs on stochastic noise to be quantified in a high-throughput manner.

## Materials and Methods

### Fitting a damped sinusoid to experimental data

Raw experimental data *x*(*t*_*i*_), *i* ∈ {0, …, *N* − 1} are first detrended using Hodrick-Prescott filter with a smoothing parameter $\gamma =0.05{\left(\frac{24\;\text{hrs}}{T_{s}}\right)}^{4}$, in which *T*_*s*_ is the sampling rate (in hours) [24]. The detrended data are then filtered using a low-pass filter to remove high-frequency noise (forward-backward Butterworth filter with *n* = 5, *w*_*c*_ = 0.1). We denote the detrended and filtered experimental data by *y*(*t*_*i*_). A damped sinusoid, specified by:

$$\begin{array}{c} \hat{y}\left(t\right)=Ae^{-dt}\sin(\frac{2\pi t}{T}+\theta ) \end{array}$$

is then fit to the filtered data. For numerical efficiency, the period, *T*, and damping rate, *d*, parameters are fit first using a matrix pencil method [25], reviewed in [26]. Amplitude, *A*, and phase, *θ*, parameters are subsequently fit using a linear least-squares regression. Note that in this manuscript we use *amplitude* to denote the initial rhythm strength, and *damping rate* to denote the rate at which this strength decays with time. Overall *R* ^2^ values for the regression were calculated from the residual error between the detrended data and fitted sinusoid:

$$\begin{array}{c} R^{2}=1-\frac{\sum_{i=0}^{N-1}{\left(y\left(t_{i}\right)-\hat{y}\left(t_{i}\right)\right)}^{2}}{\sum_{i=0}^{N-1}{\left(y\left(t_{i}\right)-\bar{y}\left(t_{i}\right)\right)}^{2}} \end{array}$$

in which $\bar{y}(t_{i})$ represents the mean of the detrended data.

### Processing single-cell bioluminescence data

Single-cell bioluminescence data for 79 cells was obtained from Leise *et al*., 2012 [22]. As was done in the original study, a discrete wavelet transform (using PyWavelets, http://www.pybytes.com/pywavelets) was performed to detrend and remove noise. A discrete wavelet transform decomposes the signal into multiple frequency bands [27]. By only considering frequency bands close to the circadian frequency, high-frequency noise and low-frequency baseline oscillations can be removed.

#### Sorting cells by noise level

As in the original study, various parameters describing the average noise level of each cell were collected. Traces were denoised and detrended by keeping only the (8hr,258hr) wavelet components—resulting in rhythms that only contained oscillations with periods between 8 and 258 hours. From these smoothed trajectories, a Hilbert transform was used estimate points at which the phase crossed 0 to find period and amplitude coefficients of variation. An additional noise parameter, the standard deviation in the (1hr,8hr) wavelet components divided by the overall rhythm amplitude, was used to quantify the high-frequency noise of the system. This wavelet component was chosen as it contained only the highest-frequency noise in the signal’s spectrum. From these three noise variables, a combined noise metric was constructed by projecting the variables along their first principle component (using scikit-learn, http://scikit-learn.org/). Cells were ranked according to this combined noise metric, and a high-noise group and low-noise group were constructed by taking the 39 highest-noise and lowest-noise cells, respectively. The raw bioluminescence profiles were not initially synchronized, so the traces were offset to have the same starting phase in order to simulate the gradual desynchronization of a group of oscillators. This was accomplished by starting each trace at the first phase zero-crossing, found using a Hilbert transform.

#### Bootstrap estimations of the damping rate difference

Averaged traces for low and high-noise group displayed a damped sinusoidal rhythm. The first 4 days of rhythms (*T*_*s*_ = 0.5, *N* = 192) were fit using a damped sinusoid. To ensure the difference in damping rate between groups was statistically significant, a bootstrap analysis was performed. In each of 10,000 bootstrap trials, cells were randomly assigned evenly to either the low-noise or the high-noise group (one cell was randomly omitted in each trial to ensure even group sizes). The absolute difference in damping rate between the two populations was recorded to yield a two-tailed test. The observed test statistic, ∣*d*_*h*_ − *d*_*l*_∣ = 6.65 × 10^−3^, was found to be significant at the *α* = 0.05 confidence threshold (*p* = 0.0264).

### Quantifying dose-dependent effects of small molecule modulators

Bioluminescence traces (*T*_*s*_ = 1.67, *N* = 71) with increasing small molecule concentrations were fit with a damped sinusoid using the method described in a previous section. Because the small molecules were toxic at very high concentrations, experiments were removed from further analysis where *R* ^2^ < 0.80 (S1 Fig).

### *In silico* prediction of small molecule experiments

A previously published mathematical model of circadian rhythms [28] was used to predict the effects on population damping rate from the dose-dependent small molecule experiments. The parameters used to capture the effects of each small molecule were the same as described previously [13]. The model was converted to a stochastic biochemical system and subsequently simulated using StochKit2 [29] (via GillesPy, https://github.com/JohnAbel/gillespy). Population-level rhythms were found by taking the average of 1,000 noninteracting oscillators, starting at identical initial conditions. The only parameter left unspecified by the deterministic model was the cell volume, Ω, which controlled the amount of noise in the system. For each Ω, a damped sinusoid was fit to the population-averaged state trajectory. An *R* ^2^ value was calculated for each fit, taking into account all eight state variables.

#### Fitting the volume parameter

We calculated an average experimental damping rate of *d* = 0.0151 from the 0*μM* bioluminescence trajectories for both KL001 and longdaysin. *In silico* damping rates were calculated for logarithmically spaced values of Ω ∈ (100,500). Ten independent groups of 1,000 oscillators were simulated for each Ω, from which the means and standard errors were found. Simulations in which *R* ^2^ < 0.90 were removed from further analysis. A weighted least-squares regression (using statsmodels, http://statsmodels.sourceforge.net/) was performed for log *d* vs. log O, using the log SEM of each measurement as a regression weight (S2 Fig). The best fit was found to be Ω = 226.3 ± 9.0, and was used for subsequent model simulations.

#### Parameter knockdown experiments

We replicated the effects of the small molecules KL001 and longdaysin mathematically through the reductions of the *vdcn* and *vac*1*p* parameters, respectively (S1 and S2 Tables). Knockdown simulations were performed with 20 values of each parameter, linearly spaced between 100% and 15% of their nominal value. Similar to the volume calibration simulations, 10 independent populations of 1, 000 oscillators were simulated from an initially entrained state. Means and standard errors in period and damping rate were calculated from each population-averaged trajectory. Simulations in which *R* ^2^ < 0.90 were removed from further analysis.

### Fitting the genome-wide siRNA screen

We analyzed the data and annotations for the 111,743 wells (*T*_*s*_ = 2, *N* = 72) in the Zhang *et al*., 2009 screen [12]. Fits for which the *R* ^2^ < 0.80 were discarded. The natural logarithm of the amplitude was used, since it more closely resembled a normal distribution and was on a similar scale to the damping rate. Plate to plate variation, as shown in S3 Fig, was more severe than variation on a well-to-well basis, S4 Fig. Parameters were therefore normalized on a plate-by-plate basis using a robust z-score [30]:

$$\begin{array}{c} z_{R,i}=\frac{p_{i}-M\left(p_{i}\right)}{M(|p_{i}-M\left(p_{i}\right)|)},\;i\in \left\{0,\cdots ,P-1\right\} \end{array}$$

where *M*(⋅) denotes the median of a vector, and *p*_*i*_ contains all the points in one plate, and 𝒫 is the number of plates. We removed outlier points prior to calculating the moments of the distributions, Pearson correlation coefficients, and performing the multivariable linear regression. Outliers were defined as points that contained a z-metric (in either period, phase, amplitude, or damping rate) with an absolute value greater than eight. We chose the “control” wells to be those that contained no siRNA, as these proved to be more numerous than those containing reference siRNA perturbations and were clustered similarly to the highest-density regions of the perturbed fits.

#### Detecting outlier perturbations

We found the average response to each gene perturbation by grouping the perturbed dataset by target gene ID (using pandas, http://pandas.pydata.org/). A Hotelling’s *T* ^2^ test was used to determine whether the means of each gene knockdown was significantly different from the control population. While different siRNA constructs will have different knockdown efficiencies, grouping based on gene target helps to eliminate the effect of off-target activities. A robust covariance estimator was used to find the location and covariance of the control distribution (using scikit-learn). Because the control distribution (*n* = 11,253) is much larger than that for any particular gene ID (*n* ≈ 4), the effect of the perturbed sample on the pooled covariance was neglected.

### Software

All computations were performed using Python. Code used to perform the analysis and produce the figures in this manuscript can be found online at https://github.com/pstjohn/decay-methods.

## Results

### Higher noise results in faster damping in population-level rhythms

**Fig 1:**
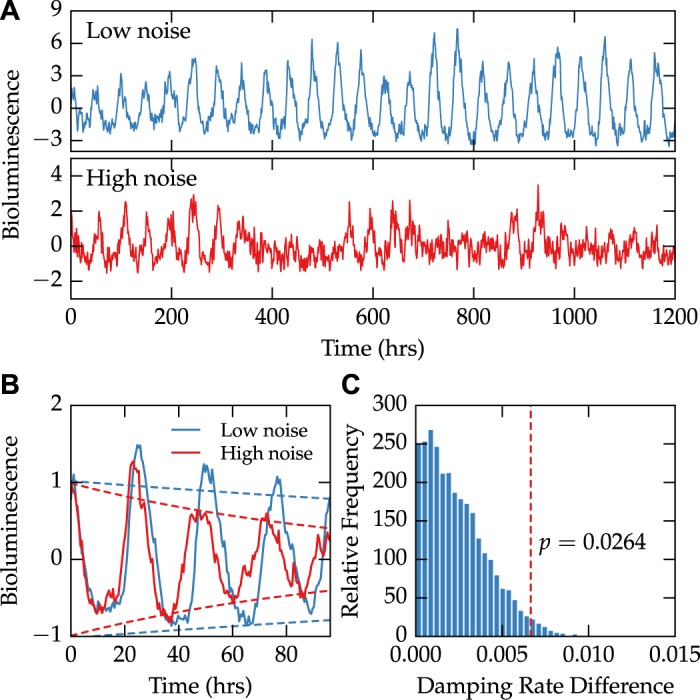
Single-cell bioluminescence recordings show that higher stochastic noise results in faster damping at the population-level. Data on the bioluminescence of single-cell fibroblasts was taken from Leise *et al*., 2012 [22]. (**A**) Cells were sorted into two groups depending on their degree of stochastic noise. An example trace from each of the two groups is shown, demonstrating different levels of noise present in the dataset. (**B**) After artificially synchronizing each cell, we calculate averaged bioluminescence rhythms of each group (solid lines). A damped sinusoid fit to both groups reveals a difference in damping rate, demonstrated by fitted envelope functions (±*A*exp−*dt*, dashed lines). (**C**) The observed absolute difference in damping rate was shown to be significant (*p* = 0.0264) using 10,000 bootstrap trials.

While both intrinsic and extrinsic noise sources can contribute to population-level damping, intrinsic noise is thought to play a more significant role in circadian systems [2]. We therefore first sought to determine whether changes to intrinsic stochastic noise alone are sufficient to explain population-level changes in damping rate. To do this, we calculated noise characteristics from experimental data on individual PER2::LUC fibroblast cells [22]. Cells were sorted into two groups, a low-noise group and a high-noise group, based on the relative high-frequency noise, period variability, and amplitude variability present in each trace. Example rhythms from cells in both groups are shown in Fig 1A. Because the cells were not synchronized at the start of the recording, this effect is replicated *in silico* by shifting each series in time to align their start phases. Population-level bioluminescence traces were then found by averaging the cellular PER2::LUC signal in each group. Both populations displayed averaged rhythms that resembled a damped sinusoid, similar to those seen in bioluminescence recordings of entire cell cultures. Fitting the averaged expression of each group with a damped sinusoid revealed that the low-noise group also had a lower damping rate (Fig 1B). The significance of this difference was confirmed via a bootstrap analysis (Fig 1C), where cells were randomly assigned in each bootstrap trial to either the low-noise or the high-noise group.

### Clock perturbations can change single-cell stochastic noise

#### A small molecule causes dose-dependent changes in the population-level damping rate of cultured cells

**Fig 2:**
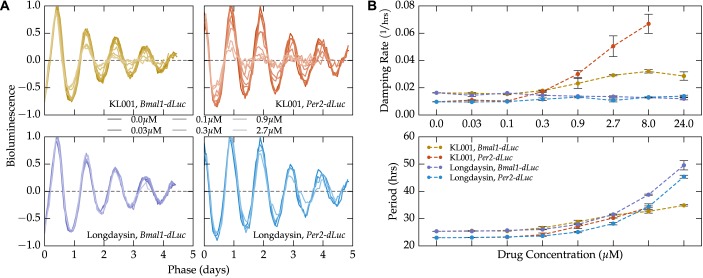
Small molecule modulator KL001 increases damping rate in a dose-dependent fashion. Experimental data on the dose-dependent effects of small molecules KL001 and longdaysin on cultured circadian reporter cells was taken from Hirota *et al*., 2012 [28]. (**A**) Detrended bioluminescence signals from the two reporter systems and two small molecules are shown normalized by the fitted amplitude, period, and phase. The normalized bioluminescence highlights the dose-dependent change in damping rate seen with the KL001 application (top), but not with longdaysin (bottom). (**B**) Quantification of the dose-dependent change in damping rate caused by small molecule modulators. While both molecules lead to a dose-dependent increase in period, only KL001 shows a reliable change in damping rate.

We next demonstrate that perturbations to the transcriptional oscillator are capable of altering population-level damping rate. The actions of small-molecule circadian modulators KL001 and longdaysin are well-characterized, and are known to affect circadian period and amplitude in a dose-dependent fashion [13]. By fitting experimental data on the population-level responses to increasing dosages of each molecule with a damped sinusoid, we show that KL001, but not longdaysin, increases damping rate in a dose-dependent fashion (Fig 2, S1 Fig). This change in damping rate is consistent across both reporter systems (*Bmal1-dLuc* and *Per2-dLuc* U2OS cells), indicating it is a fundamental property of the overall gene regulatory network. This result indicates that stochastic noise can be altered by perturbations known to affect the transcription-translation feedback loop.

#### Mathematical model predicts KL001 damping rate increase comes from increased single-cell stochastic noise

We next test the hypothesis that the dose-dependent changes in damping rate from KL001 is due to changes in intrinsic (cell-autonomous) noise characteristics. To do this, we employed a mathematical model of circadian rhythms previously used to explain the effects of both small molecule perturbations [13], summarized in S1 and S2 Tables. In order to capture changes to noise characteristics, we first converted the model to a stochastic biochemical system. Population-level rhythms were generated by averaging the trajectories of 1,000 individual, noninteracting oscillators. The only free parameter in converting the existing deterministic model to a stochastic one is the cell volume, which was determined by fitting the observed population-level damping rate to that of the experimental control traces (S2 Fig).

**Fig 3:**
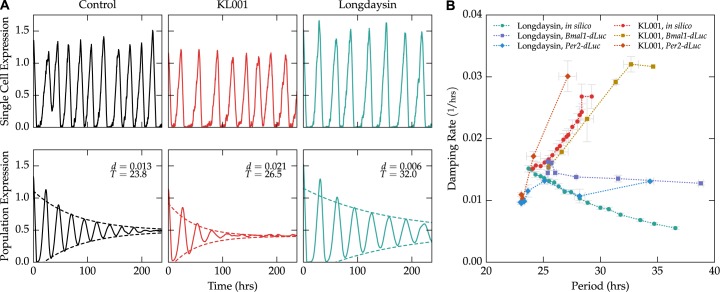
Mathematical model accurately predicts dose-dependent changes in damping rate. (**A**) Example single-cell trajectories (top) and population-averaged trajectories (bottom, mean of 1,000 cells) of cells under various treatments. Cells with the nominal parameter set (left, black) closely match the experimental damping rate for unperturbed cells. Cells with simulated KL001 action (red, center) are noisier at the single-cell level, and show faster damping at the population-level. Cells with simulated longdaysin action show slightly more accurate single-cell rhythms, with a corresponding reduction in the population-level damping rate. (**B**) The model accurately predicts the general trend of period vs. damping rate for both KL001 and longdaysin perturbations. Experimental data points represent the mean of two replications at each concentration. Computational data points represent the mean of ten independent population simulations.

The model was then used to predict the effects of KL001 and longdaysin administration on single-cell noise and population damping rates (Fig 3A). Reductions in parameters previously attributed to the activities of each small molecule caused dose-dependent changes in period and damping rate at the population-level that closely matched experimental results (Fig 3B). As the model includes no cell-to-cell communication or heterogeneity, this difference is manifested solely by changing the noise characteristics of individual cells. This quantitative prediction by the mathematical model lends further support to the assumptions that individual cells are independent and sustained oscillators, and intrinsic noise plays the dominant role in determining population-level damping.

### Genome-wide effects of siRNA knockdown on single-cell stochastic noise

**Fig 4:**
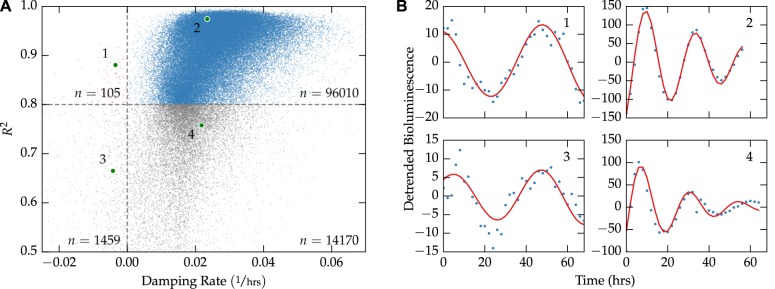
Fit quality vs. damping rate for the genome-wide siRNA screen. (**A**) A plot of the 111,743 individual fits shows that the majority of fits have a high *R* ^2^ value and positive damping rate. Only fits with *R* ^2^ > 0.8 were kept for further analysis. (**B**) Examples chosen randomly from each of the four quadrant regions in (A). Sinusoidal parameters for fits 1–2 can be more confidently inferred than those for fits 3–4.

Unlike from using single-cell imaging, inferring stochastic noise from the desynchronization rate of population-level recordings can be applied to existing and future high-throughput circadian screens. We demonstrate the insights that can be gained from such an approach by analyzing the publicly available genome-wide siRNA screen from Zhang *et al*., 2009 [12]. The results of fitting a damped sinusoid to each of the 111,743 bioluminescence trajectories is shown in Fig 4, in which 86% of fits had an *R* ^2^ > 0.8. Since sinusoidal parameters can only be confidently inferred for fits in which the *R* ^2^ was sufficiently high, wells were removed from further analysis if *R* ^2^ < 0.8. Additionally, of the fits with a high *R* ^2^ value, only a small minority (0.1%) had a negative damping rate. This trend supports the assumption that intercellular synchronization is unlikely in cultured U2OS cells.

**Fig 5:**
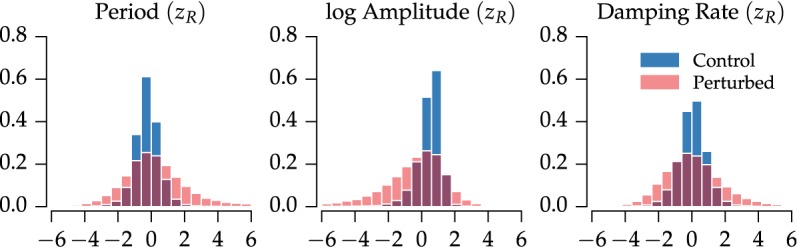
Distributions in fitted parameters for the genome-wide siRNA screen. For each well in the high-throughput screen, the period, amplitude and damping rate are calculated. After normalization, distributions in robust z-scores closely resemble normal distributions. For all parameters, the region of highest density is consistent between the control and perturbed populations, indicating many perturbations do not appreciably change clock dynamics.

We next checked how parameters varied on a plate-to-plate and well-to-well basis. Well-to-well variation was relatively absent, aside from expected variation due to long- and short-period controls (S4 Fig). Fits were normalized to remove plate-to-plate variation (S3 Fig) using a robust z-score [30]. Additionally, we separated wells into a “perturbed” category and “control” category, depending on whether or not the well contained an siRNA perturbation. As we show in Fig 5, all fitted parameters displayed normal-like distributions, in which the control distributions showed much tighter clustering around the most likely values. Quantifications of the mean, variance, skewness, and kurtosis for each distribution are shown in Table 1.

**Table 1:** Moments of the fitted parameter distributions (Fig 5) after normalization and outlier removal. Parameters were normalized by subtracting the median and dividing by the median absolute deviation on a plate-by-plate basis. Control distributions had less variance, were less skewed, and were more peaked then their perturbed counterparts.

	*T*		ln *A*		*d*	
	C	P	C	P	C	P
Mean	−0.234	0.187	0.443	−0.343	0.043	0.090
Variance	0.599	3.313	0.605	3.074	0.771	2.850
Skewness	0.153	0.367	−1.823	−0.580	−0.107	0.371
Kurtosis	3.772	0.591	8.329	0.476	2.423	0.373

#### Damping rate is independent of other sinusoidal parameters

Since an siRNA’s effect on period length does not effectively predict its effect on amplitude or phase (and vice-versa), we hypothesized that damping rate would similarly be independently affected by siRNA perturbations. Low Pearson correlation coefficients between normalized parameter distributions defend this hypothesis, with the highest correlations among variables seen between amplitude and damping rate (*ρ* = 0.285, Table 2). Additionally, a multivariate linear regression of damping rate as a function of period, amplitude, phase, and perturbation type (control or perturbed, a categorical variable) produced an *R* ^2^ value of only 0.169 (S4 Table). These results reinforce the notion that the population-level damping rate describes an independent feature of the underlying oscillator.

**Table 2:** Correlation among normalized parameters of the high-throughput siRNA screen. Pearson correlation coefficients are relatively low between fitted parameters, indicating that changes to damping rate (and thereby stochastic noise) are not explained by changes to period, amplitude, or phase.

	*d*	ln *A*	*T*	*θ*
*d*	1.000	0.285	−0.142	−0.269
ln *A*	0.285	1.000	−0.022	−0.112
*T*	−0.142	−0.022	1.000	−0.113
*θ*	−0.269	−0.112	−0.113	1.000

#### The unperturbed clock lies at the Pareto frontier of high amplitude and low noise

**Fig 6:**
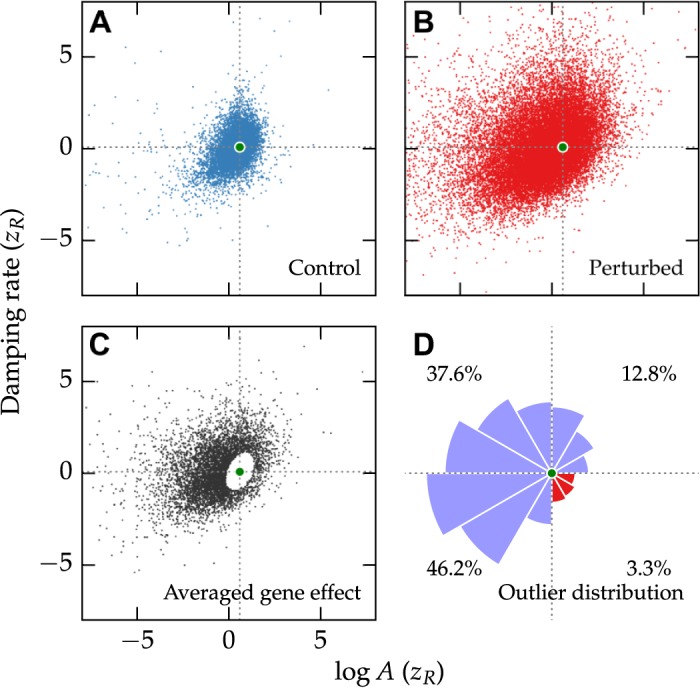
Effects of siRNA knockdowns on amplitude and damping rate. Clock robustness is a function of both amplitude and damping rate. Distributions in amplitude and damping rate for control wells (**A**) or perturbed wells (**B**) indicate that perturbations tend to shift the clock towards regions of higher damping rate or lower amplitude. Green dots in each figure indicates the mean of the control population. (**C**) Averaged effect of siRNA knockdown after grouping the perturbed population by Gene ID. Only those genes that were significantly different from the control distribution are shown (Hotelling’s *T* ^2^ test, *α* = 0.01). (**D**) Radial histogram of the significant gene perturbations shown in (C). The area of each slice is proportional to the frequency of perturbations away from the mean in that direction. Very few gene knockdowns result in both higher amplitude and lower damping rate (red slices, lower right quadrant).

The ability of a population of oscillators to maintain robust oscillations is a function both of its initial amplitude as well as its damping rate. A robust oscillator is therefore one with high initial amplitude and low stochastic noise. Scatter plots of the control and perturbed distributions are shown in Fig 6A and 6B, which indicate that few outlier points reside in the lower damping rate, higher amplitude quadrant. In order to find perturbations that confidently change robustness, we grouped the siRNA perturbations by target gene and performed a two-sample Hotelling’s *T* ^2^ test against the control population. The resulting significant gene perturbations (75% of all genes) are shown in Fig 6C. Quantifying the distribution in outliers by quadrant, it is clear that only a small minority of perturbations (3.3%) simultaneously increase amplitude and decrease stochastic noise. It therefore appears that the unperturbed clock optimizes some trade-off between high amplitude and low stochastic noise, such that it is unlikely the knockdown of a single gene will make oscillations more robust.

### Effect of cell heterogeneity

In the preceding sections, we have demonstrated that changes to single-cell intrinsic noise are sufficient to explain the observed changes in population-level damping. However, experimental work has shown that cell-autonomous fibroblast cells have a distribution of mean free-running periods [22]. Indeed, prior to the availability of single-cell data, studies explored the possibility that differences in mean periods served as the mechanism behind population-level damping [31]. While it is true that the dephasing of a group of oscillators can be caused by both variance in the mean period as well as cycle-to-cycle variability, intrinsic stochastic noise may play a more significant role. We show that there is greater variance in period on a cycle-to-cycle basis than on a cell-to-cell basis in cultured fibroblast cells (S5 Fig): individual cell period lengths have an average inner quartile range (IQR) of 3.18 hrs, while cell-to-cell average period has an IQR of 1.55 hrs. These results are independently confirmed by a previous study using Bayesian modeling, which found a standard deviation of periods within cells of 1.43 hrs, and 0.89 hrs across the population [32]. A similar result has also been observed in dispersed SCN neurons, suggesting that while both intrinsic and extrinsic period heterogeneity likely contribute to the dephasing kinetics, cell-to-cell differences are less severe than cell-autonomous noise [2].

It is also possible that damping rate changes due to siRNA or small molecule perturbation could be manifested through altering the system’s extrinsic noise. Such a change could be caused by an unequal uptake of siRNA or drug on a cell-to-cell basis, as has been demonstrated by a distribution of single-cell knockdown efficiency through flow cytometry [33]. This effect would increase cell heterogeneity and lead to faster dephasing kinetics. While differentiating between intrinsic and extrinsic noise sources from population-level data is possible in theory (S6 Fig), these differences are likely not identifiable from typical population-level data (S7 Fig). This distinction would likely be possible with single-cell level data, as has been done in other experimental systems [15]. However, such an experiment would likely not be amenable to high-throughput methods. Differences in damping rates are therefore best viewed as representative of changes to overall stochastic noise from both intrinsic and extrinsic factors. Since both types of noise are important to determining the overall function of population-level rhythms, damping rates are still a valuable method of quantifying stochastic noise.

## Discussion

In this study we have shown that the damping rate of population-level circadian oscillations can be changed by genetic or pharmacological perturbations. As populations-level rhythms are determined by the coherence of many individual cells, desynchronization due to stochastic noise is a likely explanation for population-level damping. Using single-cell data, we showed that population-level damping rate is proportional to single-cell noise. Furthermore, we used a computational model to predict the changes in damping rate from two small molecules, demonstrating that changes to intrinsic stochastic noise are sufficient to explain the observed damping rate changes.

We have described a method by which changes to stochastic noise can be estimated from population-level circadian bioluminescence recordings. An overview of the computational steps involved in our method are outlined in S8 Fig. While the method can be applied to existing experimental data, there are also practical considerations for the design of future experiments. Because the damping rate must be inferred from the time-varying amplitude, collecting bioluminescence data for longer time periods yields more accurate results and reduces the potential impact of initial transient regions. Additionally, a sampling rate that is high enough to confidently capture the peaks and troughs of gene expression is required—in this study, the 2 hr sampling window of the siRNA screen proved sufficient. While achieving such a rate is typically not difficult for bioluminescence experiments, it may limit the method’s applicability in experiments where samples need to be analyzed at each time point. We also note that there are many available tools for detrending and regressing time-series data. While we prioritized computationally efficient methods (which could be scaled to genome-wide screens), the best methods for any particular application may vary depending on the data.

As high amplitude circadian oscillations are important for maintaining metabolic health, many studies have sought to find small molecule candidates that increase oscillatory amplitude [9]. In the search for circadian clock therapeutics, high-throughput methods are frequently used to screen for such drugs, often neglecting potential effects on cell-autonomous noise. While we have demonstrated a method by which the effects of small molecules on noise can be inferred from high-throughput methods, we have also shown that the potential improvement of clock robustness may be limited. Amplitudes of circadian rhythms may therefore be best increased by small molecule therapies that act transiently to synchronize peripheral oscillators. While such a method would require accurate alignment of drug administration to the correct circadian phase, a recent *in silico* study has demonstrated the potential effectiveness of such an approach in improving amplitudes in peripheral tissues [34]. Finally, the ability to extract an additional biologically relevant parameter from existing datasets will likely prove useful for many studies, as it allows further differentiation between perturbations that might otherwise have identical effects on bioluminescence rhythms.

## Supporting Information

S1 Fig Fit quality for the dose-dependent small molecule screens. The bioluminescence rhythms in both reporter systems were well-described by a damped sinusoid. As the molecules were toxic to the reporter cells at high concentrations, fit quality declined with increasing dosage. Only fits with *R* ^2^ > 0.8 were kept for further analysis.
(EPS)

S2 Fig Calibration curve for fitting the volume parameter to experimental data. The model’s volume parameter was linearly related to the population-averaged damping rate on a log-log scale. Error bars represent the standard error of the mean, calculated by 10 independent replicates for each volume. Points shown in gray had an average *R* ^2^ < 0.9 and were excluded from the linear regression. Solid and dashed grey lines indicate the mean and 95% confidence intervals of the linear regression, respectively.
(EPS)

S3 Fig Plate-to-plate variation of fitted parameters in the Zhang *et al*., 2009 genome-wide siRNA screen. Dots indicate the median of each plate, with lines extending from the 5^th^ to 95^th^ percentile. While parameter fits were of similar magnitude for all plates, some inconsistencies were present. In order to accurately compare perturbations and controls between plate experiments, we normalized fitted parameters on a plate-by-plate basis.
(EPS)

S4 Fig Well-to-well variation in the Zhang *et al*., 2009 genome-wide siRNA screen. Similar to S3 Fig, dots indicate the median value of each parameter in each well, with lines extending from the 5^th^ to 95^th^ percentile. Well position did not seem to affect the fitted values, particularly in the middle regions that contained the siRNA knockdown library. Wells on either end showed significant variation, but these are likely due to the spotting of long- and short-period controls in the same position on each plate.
(EPS)

S5 Fig Variability in period length from cycle to cycle is greater than from cell to cell. Using single-cell fibroblast data from [22], a distribution of period lengths were calculated for each cell. Average period length for each cell is shown by a blue dot, with cells sorted from lowest to highest average period. Error bars extend from the 5^th^ to 95^th^ percentile period within each cell. The gray shaded region extends from the 5^th^ to 95^th^ percentile of the average period lengths for the entire population. 87% of cells show greater variability from cycle-to-cycle than the overall period heterogeneity.
(EPS)

S6 Fig Period heterogeneity and phase diffusion display different damping profiles. (*Left*) A simulated population of homogeneous, noisy oscillators dephases due to cycle-to-cycle variability. The envelope of the averaged expression is proportional to *t*. (*Right*) A deterministic, heterogeneous population dephases due to different free-running periods. In this case, the population displays ballistic phase diffusion, in which envelope of the population-averaged expression changes proportionally to *t* ^2^. (See Rougemont & Naef, 2007 [23] for a more detailed discussion).
(EPS)

S7 FigIdentifiability of intrinsic and extrinsic noise factors in population-level data.A simple model with both intrinsic and extrinsic noise (S3 Table) was used to determine the identifiability of noise sources. Bioluminescence profiles were generated from a population of 1000 individual oscillators. The stochastic volume parameter, Ω, controls the intrinsic noise, with larger volumes resulting in more deterministic profiles. Extrinsic noise was generated by using a normal distribution of free running periods, $𝒩(1,\sigma_{T}^{2})$ days. Points represent window functions (amplitude over time) from 10 independent replications. Lines represent linear regressions, except in the right panel of part A, where a quadratic regression was used. (**A**) For models with pure intrinsic (left) or pure extrinsic (right) noise sources, amplitude damping proportional to *e* ^*dt*^ vs. *e* ^*dt*^2^^, respectively, was readily distinguishable. (**B**) For models with both intrinsic and extrinsic noise sources, it was not possible to differentiate between increasing intrinsic noise (left) and increasing extrinsic noise (right), as both showed nearly linear relationships between time and log amplitude. The reduction in consistency between points seen for very small amplitude oscillations represents a fundamental limitation of the method, as noise dominates when oscillations approach steady state.(EPS)

S8 Fig Overview of the computational method. (**A**) Raw bioluminescence traces are collected from cultured cells. Since the method is mainly dependent on amplitude over time, longer experiments yield more identifiable damping rates. (**B**) Raw traces are detrended and denoised. This can be accomplished using a variety of methods, including the discrete wavelet transform. (**C**) The window of the oscillations (amplitude vs. time) is plotted on a semi-log axis to make the exponential damping apparent. Window functions can be calculated using a Hilbert transform, or simply by using the absolute value of the local peaks (as was done here). A linear regression of the window function in semi-log space will yield both the initial amplitude (intercept) and damping rate (slope), with care being taken to avoid the over-influence of outlier points. Standard statistical techniques could then be used to verify that differences in slope are indeed significant.
(EPS)

S1 Table A model of the mammalian core circadian feedback loop, from Hirota *et al*., 2012 [28]. Lower case letters (p: *Per*, c1: *Cry1*, c2: *Cry2*) are mRNA state variables. Uppercase letters (P: PER, C1: CRY1, C2: CRY2) are the free (cytosolic) proteins. C1N: CRY1 and C2N: CRY2 are the nuclear proteins.
(PDF)

S2 Table Parameter values for the model in S1 Table. Nominal values for the kinetic parameters are shown below, as published in Hirota *et al*., 2012 [28].
(PDF)

S3 Table Simple model of transcription-translation oscillations with intrinsic and extrinsic noise. A model with both intrinsic and extrinsic noise, developed to examine the effect of each noise source on population-level amplitudes. Intrinsic noise is generated by simulating the solution stochastically using GillesPy. Extrinsic noise is generated by varying the free-running period, *t*_*c*_. Equations adapted from [35].
(PDF)

S4 Table Multivariable linear regression results. Fit statistics that demonstrate the effect of each other fitted parameter on damping rate. Of particular note is the perturbation type categorical variable, which demonstrates that the presence of siRNA perturbation increases damping rate on average, controlling for changes in other variables. Higher amplitude is also correlated with higher damping rate. However, in total damping rate is poorly predicted by the other fitted variables (*R* ^2^ = 0.169), indicating it describes an independent oscillatory feature.
(PDF)
