# Supplementary material for: Quantifying Stochastic Noise in Cultured Circadian Reporter Cells
Source: PLoS Comput Biol. 2015 Nov 20;11(11):e1004451. doi: 10.1371/journal.pcbi.1004451 (PMC4654518; doi:10.1371/journal.pcbi.1004451)
Supplement: S1 Table — Lower case letters (p: Per, c1: Cry1, c2: Cry2) are mRNA state variables. Uppercase letters (P: PER, C1: CRY1, C2: CRY2) are the free (cytosolic) proteins. C1N: CRY1 and C2N: CRY2 are the nuclear proteins. (PDF) [file pcbi.1004451.s009.pdf]

Table S1: A model of the mammalian core circadian feedback loop, from Hirota *et al.*, 2012. Lower case letters (p: *Per*, c1: *Cry1*, c2: *Cry2*) are mRNA state variables. Uppercase letters (P: PER, C1: CRY1, C2: CRY2) are the free (cytosolic) proteins. C1N: CRY1 and C2N: CRY2 are the nuclear proteins.

$$\frac{d\mathbf{p}}{dt} = \frac{v_{\text{txn},\mathbf{p}}}{k_{\text{txn},\mathbf{p}} + (\mathbf{C1N} + \mathbf{C2N})^3} - \frac{v_{\text{deg},\mathbf{p}}\ \mathbf{p}}{k_{\text{deg},\mathbf{p}} + \mathbf{p}} \tag{1}$$

$$\frac{d\mathbf{c1}}{dt} = \frac{v_{\text{txn},\mathbf{c1}}}{k_{\text{txn},\mathbf{c}} + (\mathbf{C1N} + \mathbf{C2N})^3} - \frac{v_{\text{deg},\mathbf{c1}}\ \mathbf{c1}}{k_{\text{deg},\mathbf{c}} + \mathbf{c1}} \tag{2}$$

$$\frac{d\mathbf{c2}}{dt} = \frac{v_{\text{txn},\mathbf{c2}}}{k_{\text{txn},\mathbf{c}} + (\mathbf{C1N} + \mathbf{C2N})^3} - \frac{v_{\text{deg},\mathbf{c2}}\ \mathbf{c2}}{k_{\text{deg},\mathbf{c}} + \mathbf{c2}} \tag{3}$$

$$\begin{aligned}\frac{d\mathbf{P}}{dt} &= k_{\text{tln},\mathbf{p}}\ \mathbf{p} - \frac{v_{\text{deg},\mathbf{P}}\ \mathbf{P}}{k_{\text{deg},\mathbf{P}} + \mathbf{P}} - v_{\text{a},\mathbf{CP}}\ \mathbf{P}\ \mathbf{C1} + v_{\text{d},\mathbf{CP}}\ \mathbf{C1N} \\ &\quad - v_{\text{a},\mathbf{CP}}\ \mathbf{P}\ \mathbf{C2} + v_{\text{d},\mathbf{CP}}\ \mathbf{C2N}\end{aligned} \tag{4}$$

$$\frac{d\mathbf{C1}}{dt} = \mathbf{c1} - \frac{v_{\text{deg},\mathbf{C1}}\ \mathbf{C1}}{k_{\text{deg},\mathbf{C}} + \mathbf{C1}} - v_{\text{a},\mathbf{CP}}\ \mathbf{P}\ \mathbf{C1} + v_{\text{d},\mathbf{CP}}\ \mathbf{C1N} \tag{5}$$

$$\frac{d\mathbf{C2}}{dt} = \mathbf{c2} - \frac{v_{\text{deg},\mathbf{C2}}\ \mathbf{C2}}{k_{\text{deg},\mathbf{C}} + \mathbf{C2}} - v_{\text{a},\mathbf{CP}}\ \mathbf{P}\ \mathbf{C2} + v_{\text{d},\mathbf{CP}}\ \mathbf{C2N} \tag{6}$$

$$\frac{d\mathbf{C1N}}{dt} = -\frac{v_{\text{deg},\mathbf{CP}}\ \mathbf{C1N}}{k_{\text{deg},\mathbf{CP}} + \mathbf{C1N} + \mathbf{C2N}} + v_{\text{a},\mathbf{CP}}\ \mathbf{P}\ \mathbf{C1} - v_{\text{d},\mathbf{CP}}\ \mathbf{C1N} \tag{7}$$

$$\frac{d\mathbf{C2N}}{dt} = -\frac{\left(v_{\text{deg},\mathbf{CP}}\ m_{\mathbf{C2N}}\right)\ \mathbf{C2N}}{k_{\text{deg},\mathbf{CP}} + \mathbf{C2N} + \mathbf{C1N}} + v_{\text{a},\mathbf{CP}}\ \mathbf{P}\ \mathbf{C2} - v_{\text{d},\mathbf{CP}}\ \mathbf{C2N} \tag{8}$$
