# Supplementary material for: Quantifying Stochastic Noise in Cultured Circadian Reporter Cells
Source: PLoS Comput Biol. 2015 Nov 20;11(11):e1004451. doi: 10.1371/journal.pcbi.1004451 (PMC4654518; doi:10.1371/journal.pcbi.1004451)
Supplement: S2 Table — Nominal values for the kinetic parameters are shown below, as published in Hirota et al., 2012 [28]. (PDF) [file pcbi.1004451.s010.pdf]

Table S2: Parameter values for the model in Table S1. Nominal values for the kinetic parameters are shown below, as published in Hirota *et al.*, 2012 [15].

	Parameter	Description	Value
1	$v_{\text{txn},\mathbf{p}}$	*Per* Transcription rate	0.195
2	$v_{\text{txn},\mathbf{c1}}$	*Cry1* Transcription rate	0.131
3	$v_{\text{txn},\mathbf{c2}}$	*Cry1* Transcription rate	0.114
4	$k_{\text{txn},\mathbf{p}}$	*Per* Repression constant	0.425
5	$k_{\text{txn},\mathbf{c}}$	*Cry1/2* Repression constant	0.259
6	$v_{\text{deg},\mathbf{p}}$	*Per* Max degradation rate	0.326
7	$v_{\text{deg},\mathbf{c1}}$	*Cry1* Max degradation rate	0.676
8	$v_{\text{deg},\mathbf{c2}}$	*Cry2* Max degradation rate	0.608
9	$k_{\text{deg},\mathbf{p}}$	*Per* Degradation constant	0.011
10	$k_{\text{deg},\mathbf{c}}$	*Cry1/2* Degradation constant	1.149
11	$v_{\text{deg},\mathbf{P}}$	Max PERc degradation rate	2.970
12	$k_{\text{deg},\mathbf{P}}$	PERc degradation constant	0.034
13	$v_{\text{deg},\mathbf{C1}}$	Max CRY1c degradation rate	1.523
14	$v_{\text{deg},\mathbf{C2}}$	Max CRY2c degradation rate	1.686
15	$k_{\text{deg},\mathbf{C}}$	CRYc degradation constant	2.017
16	$v_{\text{deg},\mathbf{CP}}$	CRYn degradation rate	0.101
17	$m_{\mathbf{C2N}}$	CRY2n degradation multiplier	3.318
18	$k_{\text{deg},\mathbf{CP}}$	CRYn degradation constant	0.053
19	$v_{\text{a},\mathbf{CP}}$	CRYn association rate	0.041
20	$v_{\text{d},\mathbf{CP}}$	CRYn dissociation rate	0.002
21	$k_{\text{tln},\mathbf{p}}$	PER translation rate	3.000
