# Supplementary material for: Quantifying Stochastic Noise in Cultured Circadian Reporter Cells
Source: PLoS Comput Biol. 2015 Nov 20;11(11):e1004451. doi: 10.1371/journal.pcbi.1004451 (PMC4654518; doi:10.1371/journal.pcbi.1004451)
Supplement: S3 Table — A model with both intrinsic and extrinsic noise, developed to examine the effect of each noise source on population-level amplitudes. Intrinsic noise is generated by simulating the solution stochastically using GillesPy. Extrinsic noise is generated by varying the free-running period, t c. Equations adapted from [35]. (PDF) [file pcbi.1004451.s011.pdf]

Table S3: Simple model of transcription-translation oscillations with intrinsic and extrinsic noise. A model with both intrinsic and extrinsic noise, developed to examine the effect of each noise source on population-level amplitudes. Intrinsic noise is generated by simulating the solution stochastically using GillesPy. Extrinsic noise is generated by varying the free-running period, t_c. Equations adapted from [26].

$$\frac{dX}{dt} = t_c \left(\frac{1}{1+Y} - X \right)$$

$$\frac{dY}{dt} = t_c \left(k_t \, X - k_d \, Y - \frac{Y}{\alpha_0 + \alpha_1 \, Y + \alpha_2 Y^2} \right)$$

Parameter	Value
k_t	20
k_d	1
P	4 (or 2)
α_0	0.005
α_1	0.05
α_2	0.1
t_c	3.516
