# Supplementary material for: Quantifying Stochastic Noise in Cultured Circadian Reporter Cells
Source: PLoS Comput Biol. 2015 Nov 20;11(11):e1004451. doi: 10.1371/journal.pcbi.1004451 (PMC4654518; doi:10.1371/journal.pcbi.1004451)
Supplement: S4 Table — Fit statistics that demonstrate the effect of each other fitted parameter on damping rate. Of particular note is the perturbation type categorical variable, which demonstrates that the presence of siRNA perturbation increases damping rate on average, controlling for changes in other variables. Higher amplitude is also correlated with higher damping rate. However, in total damping rate is poorly predicted by the other fitted variables (R 2 = 0.169), indicating it describes an independent oscillatory feature. (PDF) [file pcbi.1004451.s012.pdf]

Table S4: Multivariable linear regression results. Fit statistics which demonstrate the effect of each other fitted parameter on damping rate. Of particular note is the perturbation type categorical variable, which demonstrates that the presence of siRNA perturbation increases damping rate on average, controlling for changes in other variables. Higher amplitude is also correlated with higher damping rate. However, in total damping rate is poorly predicted by the other fitted variables ($R^2 = 0.169$), indicating it describes an independent oscillatory feature.

**Dep. Variable:**	Damping Rate	**R-squared:**	0.169
**Model:**	OLS	**Adj. R-squared:**	0.169
**Method:**	Least Squares	**F-statistic:**	4782.
**Date:**	Wed, 11 Feb 2015	**Prob (F-statistic):**	0.00
**Time:**	16:26:22	**Log-Likelihood:**	$-1.7248e+05$
**No. Observations:**	94053	**AIC:**	$3.450e+05$
**Df Residuals:**	94048	**BIC:**	$3.450e+05$

	**coef**	**std err**	**t**	**P>|t|**	**[95.0% Conf. Int.]**
**intercept**	−0.0370	0.014	−2.572	0.010	−0.065, −0.009
**amplitude**	0.2375	0.003	86.282	0.000	0.232, 0.243
**period**	−0.1521	0.003	56.798	0.000	−0.157, −0.147
**phase**	−0.2354	0.003	85.598	0.000	−0.241, −0.230
**perturbation type**	0.3197	0.015	20.664	0.000	0.289, 0.350

**Omnibus:**	9769.391	**Durbin-Watson:**	1.876
**Prob(Omnibus):**	0.000	**Jarque-Bera (JB):**	18459.719
**Skewness:**	0.697	**Prob(JB):**	0.00
**Kurtosis:**	4.664	**Cond. No.**	8.34
